# Cannabis Use and Hypomania in Young People: A Prospective Analysis

**DOI:** 10.1093/schbul/sbx158

**Published:** 2017-11-28

**Authors:** Steven Marwaha, Catherine Winsper, Paul Bebbington, Daniel Smith

**Affiliations:** 1 Unit of Mental Health and Wellbeing, Warwick Medical School, University of Warwick, Coventry, UK; 2 Affective Disorders Service, Caludon Centre, Coventry, UK; 3 Division of Psychiatry, University College London, London, UK; 4 University of Glasgow, Gartnavel Royal Hospital, Glasgow, UK

**Keywords:** cannabis, mania, psychopathology, birth cohort, child abuse, ALSPAC, adolescents

## Abstract

**Background:**

Cannabis use in young people is common and associated with psychiatric disorders. However, the prospective link between cannabis use and bipolar disorder symptoms has rarely been investigated. The study hypothesis was that adolescent cannabis use is associated with hypomania in early adulthood via several potential etiological pathways.

**Methods:**

Data were used from the Avon Longitudinal Study of Parents and Children, a UK birth cohort study. The prospective link between cannabis use at age 17 and hypomania at age 22–23 years was tested using regression analysis, adjusted for gender, early environmental risk factors, alcohol and drug use, and depression and psychotic symptoms at age 18 years. Path analysis examined direct and indirect effects of the link and whether gender, childhood family adversity, or childhood abuse are associated with hypomania via an increased risk of cannabis use.

**Results:**

Data were available on 3370 participants. Cannabis use at least 2–3 times weekly was associated with later hypomania (OR = 2.21, 95% CI = 1.49–3.28) after adjustment. There was a dose–response relationship (any use vs weekly). Cannabis use mediated the association of both childhood sexual abuse and hypomania, and male gender and hypomania. The cannabis use-hypomania link was not mediated by depression or psychotic symptoms.

**Conclusions:**

Adolescent cannabis use may be an independent risk factor for future hypomania, and the nature of the association suggests a potential causal link. Cannabis use mediates the link between childhood abuse and future hypomania. As such it might be a useful target for indicated prevention of hypomania.

## Introduction

Cannabis is one of the most commonly used illegal substances of abuse in western countries. Problematic use in the general population is as high as 9.5% in the United States,^1^ while 2.6% of the UK population report having been cannabis dependent in the last year.^2^ Its use is particularly common in young people.^1^ A recent study reported that 31% of young people have used cannabis 3 or more times by age 18 years.^3^ Cannabis use is linked to multiple harms, including cardiovascular disease, motor vehicle accidents, and mental health problems. Younger people are especially vulnerable to the harmful effects of cannabis use.^4^ In adolescents, it is linked to future expression of psychotic symptoms.^5^ Though apparent, the association with depression and anxiety is weaker.^6,7^ The prospective association between cannabis use and manic symptoms or bipolar disorder diagnosis is under-investigated, though converging evidence indicates its possible importance. First, there are high rates of co-occurrence of substance misuse and bipolar disorders^8,9^ (cannabis is the most frequently used drug in bipolar disorder). Second, cannabis use in bipolar disorder is known to be associated with worse treatment compliance^10^ and more hospitalizations.^11^ Third there is a cross-sectional link between substance misuse and adolescent-onset bipolar disorder.^12^ Finally bipolar disorder in adulthood may itself be associated with increasing cannabis use.^13^

Despite these multiple associations, only prospective studies can adequately establish whether cannabis use may be causally linked to manic symptoms or bipolar disorder. In a systematic literature review covering adults over 18 years of age, we identified 6 prospective studies examining this question. We concluded that cannabis use worsens manic symptoms in those with pre-existing bipolar disorder, and might also contribute to the genesis of manic-type symptoms in those with no pre-existing bipolar disorder.^14^ In one particularl relevant study, Tijssen et al^15^ examined the 8-year longitudinal impact of substance misuse in 705 adolescents aged 14–17 years. This sampling reduced the risks of reverse causality, given that most bipolar disorder occurs after this, with a peak incidence at age 21 years.^16^ Cannabis use led to a 4-fold increase in the odds of subthreshold manic symptoms, though adjustment for psychotic symptoms was not possible. Thus, the direction and specificity of the association remains unclear (ie, it is plausible that observed links were attributable to co-morbid psychotic symptoms).

A related question concerns the mechanisms by which cannabis use may lead to manic symptoms. Cannabis use may be an independent risk factor, or a mediating factor linking earlier risk exposures (eg, childhood abuse) or characteristics of the individual (eg, sex) to the expression of bipolar symptoms. Whilst epidemiological research has begun to substantiate a mediating role for cannabis use in linking childhood trauma with adult psychosis,^17^ there has been virtually no investigation of parallel mechanisms in relation to bipolar disorder. One large cross-sectional study of adults has suggested an additive effect of childhood abuse and cannabis use on the clinical expression of bipolar disorder.^18^

In summary, prospective studies examining the association between cannabis use and hypomanic or manic symptomatology are few, and are focused on adult samples. Psychotic symptoms are frequently not controlled for, a serious limitation given significant evidence connecting cannabis and psychosis. Prospective studies examining this connection in the peak period of cannabis use and onset of hypomania are scarce, even though this is when potential interventions might be most effective.

Our main aim was to test the hypothesis that adolescent cannabis use is prospectively associated with hypomania in young adulthood. We used path analytical methods to test several potential pathways simultaneously. Specifically, we addressed the following questions: (*a*) Is cannabis use independently associated with hypomania in early adulthood following adjustment for gender, early environmental risk factors, other drug use, and depression and psychotic symptoms at age 18 years? (*b*) Do males and females follow different pathways to hypomania depending on cannabis use? (*c*) Is exposure to childhood family adversity or abuse (physical, sexual) associated with hypomania via increased likelihood of cannabis use?

## Methods

### Subjects

The Avon Longitudinal Study of Parents and Children (ALSPAC) is a UK birth cohort examining the determinants of development, health, and disease. The study has been described in detail elsewhere.^19^ ALSPAC recruited pregnant women in Avon with expected dates of delivery between the April 1, 1991 and December 31, 1992. Fourteen thousand five hundred forty-one pregnant women were initially enrolled in the study, and had returned at least one questionnaire or attended a “Children in Focus” clinic by the July 19, 1999. Of these initial pregnancies, there were 14676 foetuses, resulting in 14062 live births, of which 13988 children were alive at 1 year of age. When the oldest children were approximately 7 years old, the sample was bolstered with eligible cases who had failed to join the study originally. Consequently, when considering variables collected from age 7 onwards there are data available on 14701 children. The study website contains details of all the data that is available through a fully searchable data dictionary (http://www.bris.ac.uk/alspac/researchers/data-access/data-dictionary/). From the first trimester of pregnancy parents completed postal questionnaires about the study children’s health and development. In addition, children attended annual assessment clinics, including face-to face interviews, psychological, and physical tests. Ethical approval was obtained from the ALSPAC Law and Ethics committee and the Local Research Ethics Committees.

The representativeness of ALSPAC target population was assessed during the planning stage of the study by comparing the characteristics of children in the Avon area to 13, 135 children in the Child Health and Education Study. The Avon target population was considered to be relatively representative of the whole of Great Britain.^20^

### Assessments

Study participants were invited to complete a comprehensive postal questionnaire when they were 22–23 years old. This assessment includes the Hypomania Checklist Questionnaire (HCL-32), a self-report measure of lifetime experience of manic symptoms.^21^ Respondents are asked to consider a time when they were in a “high or hyper” condition and endorse statements about their emotions, thoughts, and behavior during this time. There are 32 symptom statements. While the HCL-32 was developed as a screening instrument for people with bipolar disorder type II in people with recurrent depressive disorders, it is also a valid and sensitive tool for young, nonclinical populations.^22^ In line with previous work,^23^ we constructed a variable to represent lifetime history of hypomania. Those with a symptom score of 14 or more (out of 32) were classed as having hypomania if they *also* reported at least one incident of “negative consequences” or of “negative plus positive consequences,” as a result of hypomanic symptoms; *and* that mood changes caused a reaction (either neutral, negative, or negative and positive) in close others; *and* that symptoms lasted for a duration of “2–3 days” or more. This was chosen because the DSM 4-day threshold excludes many individuals with bipolar disorder II^23^ and fits the ICD-10 requirement for hypomania to have lasted for a “few days.”

Cannabis use was assessed when participants were 17 years’ old. Young people were asked whether they had ever used cannabis (yes/no) and the frequency of use in the last 12 months (ie, monthly or less, 2–4 times monthly, 2–3 times weekly, 4+ times weekly). We constructed 2 cannabis use variables. The first represented any cannabis use (no/yes). The second was constructed to represent frequent (at least 2–3 times weekly cannabis use), vs infrequent or no cannabis use (ie, cannabis use less than weekly = 0; cannabis use 2–3 times or 4+ times weekly = 1) in line with previous studies.^24^ No direct information was available on duration of use other than in the last year.

Multiple family risk factors were assessed using the Family Adversity Index (FAI) during pregnancy (“long index”), 2 years (“long index”), and 4 years (“short index”). The FAI “long index” comprises 18 items, eg, maternal affective disorder, financial difficulties. The short index has the same items, but excludes social, practical, and financial support. If an adversity item was reported, it was given one point. Points were summed at each time-point for a total FAI score across the 3 time-points.

As there are robust associations of physical^25^ and sexual^26^ abuse with mental illness, we included abuse as a confounder. Physical and sexual abuse were reported by the mother via postal questionnaire when children were 1.5, 3.5, 4.8, 5.8, and 6.8 years old. We coded for the presence of any physical or sexual abuse at any time-point.

Self-reported alcohol use was assessed at age 17 with the Alcohol Use Disorders Identification Test.^27^ The AUDIT comprises 10 questions with responses ranging from 0 to 4 for each question. Scores were summed and dichotomized, with a total score of 8 or more indicating hazardous alcohol use.^27^

Participants were asked about their use of stimulant (ie, cocaine, amphetamines) and hallucinogenic drugs at age 17. As these three variables were very highly correlated, we summed the responses to create a dichotomous control variable indicating use of at least one drug.

Psychotic symptoms were assessed at age 18 using the validated Psychosis Like Symptom Interview. The interview incorporates 12 core questions relating to key psychotic experiences occurring since age 12. These are hallucinations, delusions, and experiences of thought interference. Consistent with previous research, we coded the presence of at least one definite psychotic symptom not attributable to sleep or fever.^28^

Depression symptoms were assessed using the validated Revised Clinical Interview Schedule (CIS-R) at 17–18 years.^29^ The CIS-R was self-administered via computerized interview, and establishes the severity of core symptoms of depression. We constructed a dichotomous depression variable representing any ICD-10 diagnosis of depression (whether mild, moderate, or severe).

### Analysis

As a substantial proportion of the original sample was lost to follow-up, we conducted logistic regressions to identify significant predictors of attrition. Adolescents lost to attrition were more often boys, of low birth-weight, and exposed to family adversity and frequent maternal shouting (see supplementary table S1 available online). Using the variables associated with selective dropout as the predictors, we fitted a logistic regression model (nonresponse vs response outcome) to determine weights for each individual using the inverse probability of response. We use this weighting variable in the logistic regression analyses.

We conducted unadjusted and adjusted logistic regressions (forced entry method) using SPSS version 22 to examine whether cannabis use (any and at least 2–3 times weekly) at age 17 years was associated with hypomania at 22–23 years. We used the generalized linear model (GLM) command to enable us to re-adjust the sample size following inflation from computing the inverse probability weights. We conducted the analysis in stages to test four models. Model A examined the unadjusted associations between cannabis use and hypomania. Model B examined associations following adjustment for psychotic and depressive symptoms. Model C additionally adjusted for other drug and alcohol use. Model D additionally controlled for gender, family adversity, and early childhood physical or sexual abuse.

We conducted path analysis using *Mplus version 7*. Path analysis can be used to determine whether nonexperimental data fit well with an a priori causal model allowing for the examination of direct and indirect effects of multiple independent and dependent variables.^24^

Modeled associations are unidirectional, and based on the temporal ordering of assessments (ie, earlier risk factors are hypothesized to predict later outcomes). However, because the data are nonexperimental, we cannot conclusively ascertain whether associations are causal.^30^

Using this approach, we modeled several potential causal pathways involving at least 2–3 times weekly cannabis use and hypomania, while controlling for correlations between concurrent risk factors (ie, cannabis use and other substance use) and psychopathology (ie, depression and psychotic symptoms). We also adjusted for the prospective associations between other important risk factors and hypomania (ie, other substance use, depression, and psychotic symptoms). Specifically, we modeled indirect associations between gender, family adversity, and abuse and subsequent hypomania via cannabis use. We also modeled indirect associations between cannabis use and hypomania via psychotic and depression symptoms. We modeled direct associations between cannabis and substance use, depression and psychotic symptoms, and subsequent hypomania.

Due to the very high correlations between alcohol and other drug use, we combined these 2 variables into a categorical substance use index to improve the stability and fit of the model (ie, reducing the impact of multicollinearity). We created an ordinal variable to represent *no use*, *drug or alcohol use*, and *both drug and alcohol use*.

We used probit estimation as recommended for path models with categorical outcomes.^31^ Probit regression is a log-linear approach analogous to logistic regression, producing similar chi-square statistics, *P* values and conclusions to logit models. Coefficients indicate the strength of the relationship between the predictor variable and the probability of group membership, representing the change in the probability of “case status” associated with a unit change in the independent variable. Hence, it is important to keep the scale representing the predictor in mind when interpreting results. For example, a probit coefficient of 0.031 indicates that each one-point increase in the family adversity scale resulted in an increase of 0.031 standard deviations in the predicted *Z* score of depression symptoms. Coefficients for dichotomous predictors are expected to be larger. The WLSMV estimator (weighted least squares with robust standard errors, mean, and variance adjusted) was used to yield probit co-efficients for categorical outcomes. Missing data were accommodated using the reliable full information maximum likelihood method.^32^

## Results

Data were available on 3370 participants who reported on hypomania symptoms at 22–23 years. Table 1 shows the frequencies of sociodemographic, substance use, and psychopathological variables in the final sample stratified by sex.

**Table 1.** Frequencies (or Mean Scores) of Sociodemographic, Risk, and Psychopathological Variables in the Final Sample Stratified by Sex

Variables	*N* (%)	*N* (%)
	Males	Females
Overall sample	1188 (35.3)	2182 (64.7)
Family adversity (mean; SD)	3.13 (3.31)	3.58 (3.77)
Any physical or sexual childhood abuse^a^		
No	1009 (85.9)	1919 (90.6)
Yes	165 (14.1)	198 (9.4)
Hazardous alcohol use		
No	433 (60.4)	773 (61.6)
Yes^b^	284 (39.6)	482 (38.4)
Any other drug use		
No	691 (89.6)	1199 (88.2)
Yes^c^	80 (10.4)	160 (11.8)
Weekly cannabis use		
No	735 (95.1)	1338 (98.3)
Yes	38 (4.9)	23 (1.7)
Depression		
No	793 (95.4)	1314 (89.6)
Yes	38 (4.6)	153 (10.4)
Psychotic symptoms		
No	825 (97.6)	1445 (95.8)
Yes	20 (2.4)	63 (4.2)
Hypomania		
No	1089 (91.7)	2038 (93.4)
Yes	99 (8.3)	144 (6.6)

^a^Physical or sexual abuse at any of the time-points.

^b^Defined as hazardous use according to the World Health Organization.

^c^Refers to any cocaine, amphetamines, or hallucinogenic drug use.

Alcohol and cannabis use was significantly associated: *any cannabis use*, odds ratio (OR) = 1.82; 95% CI = 1.45–2.28, and *at least 2–3 times weekly cannabis use*, OR = 2.87; 95% CI =1.68–4.91. Any and at least 2–3 times weekly cannabis use were very strongly related to other drug use (ie, amphetamines, hallucinogens, or cocaine): OR = 40.90; 95% CI = 27.41–61.03 and OR= 26.30; 95% CI = 18.21–37.97, respectively. We accordingly conducted a collinearity diagnosis. Variance inflation factors (VIFs) and tolerance values did not indicate an issue with multicollinearity in the logistic regressions.^33^

The results of the logistic regressions (weighted for the predictors of attrition) are reported in table 2. In unadjusted analysis (Model A), both *any* and *at least 2–3 times weekly* cannabis use were significantly associated with hypomania at 22–23 years, and demonstrated a dose-response relationship, ie, a stronger association for use at least 2–3 times weekly. In Model B, associations were only slightly attenuated after adjustment for psychotic and depression symptoms. In Model C, associations were further attenuated by adjustment for other drug and alcohol use, but remained robust. In Model D, associations were reduced further by adjustment for gender, family adversity, and experience of childhood abuse, but again remained significant.

**Table 2.** Unadjusted and Adjusted Associations Between Cannabis Use (Any and Weekly) and Hypomania Symptoms at 22–23 Years

Cannabis Variable	Hypomania Outcome			
	Model A	Model B, Adjustment for Psychotic Symptoms and Depression	Model C, Plus Adjustment for Other Drug and Hazardous Alcohol Use	Model D, Plus Adjustment for Gender, Family Adversity, and Early Childhood Physical or Sexual Abuse
Any cannabis use	(*N* = 1959)	(*N* = 1818)	(*N* = 1658)	(*N* = 1658)
No	[reference]	[reference]	[reference]	[reference]
Yes	**1.64 (1.37, 1.96**)	**1.66 (1.38, 1.99**)	**1.44 (1.15, 1.79**)	**1.42 (1.14, 1.77**)
Weekly cannabis use	(*N* = 1956)	(*N* = 1816)	(*N* = 1656)	(*N* = 1656)
No (infrequent cannabis use, ie, less than weekly)	[reference]	[reference]	[reference]	[reference]
Yes (at least 2–3 times weekly)	**2.80 (2.02, 3.88**)	**2.58 (1.85, 3.60**)	**2.24 (1.52, 3.31**)	**2.21 (1.49, 3.28**)

*Note:* All analyses weighted for gender, birthweight, family adversity, and maternal shouting; Model A: unadjusted associations; Model B: associations adjusted for psychotic symptoms and depression; Model C: associations adjusted for psychotic symptoms, depression, other drug use, and hazardous alcohol use; Model D: associations adjusted for psychotic symptoms and depression, other drug use, hazardous alcohol use, gender, family adversity, and childhood abuse. Bold signifies the positive results clear in the table.

Cannabis use at least 2–3 times weekly significantly predicted both depression (OR = 2.48; 95% CI=1.60–3.83) and psychotic symptoms (OR = 3.33; 95% CI = 2.01–5.53) in unadjusted analysis.

**Fig. 1.**
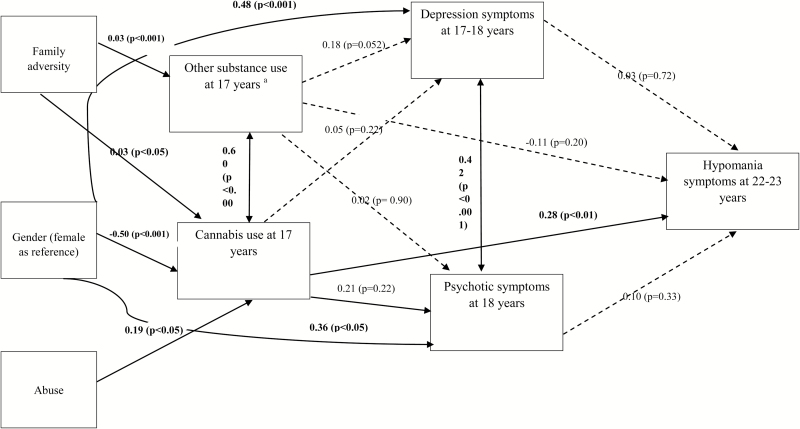
Pathways from cannabis use to hypomania. Main direct effects in the final path model. Significant pathways signified by solid arrows; nonsignificant modeled pathways represented by dotted lines; Model fit: CFI = 1.00; RMSEA = 0.00; chi-square model fit = 2.57, *P* = .92; ^a^Other substance use = alcohol and control drugs.

Path analysis fit indices indicated excellent model fit: χ^2^ = 2.57, *P* = .92; RMSEA = 0.00; CFI = 1.00. Consistent with the adjusted logistic regression analysis, cannabis use at 17 years was significantly and independently associated with subsequent hypomania in the final model controlling for all pathways. Direct associations are shown in figure 1. The association between cannabis use and hypomania was not significantly mediated by depression or psychotic symptoms. However, cannabis use significantly mediated associations of gender and abuse with subsequent hypomania (table 3).

**Table 3.** Unstandardized Probit Coefficients (β) for the Hypothesized Indirect Pathways to Hypomania Symptoms With Cannabis Use, Psychotic Symptoms, and Depression as Mediators

	Via Weekly Cannabis			Via Psychotic Symptoms			Via Depression Symptoms		
	β	SE	*P*	β	SE	*P*	β	SE	*P*
Predictor									
Gender	**−0.139**	**0.053**	**.009**						
Family adversity	0.009	0.005	.069						
Abuse	**0.052**	**0.025**	**.041**						
Weekly cannabis	N/A	N/A	N/A	0.02	0.025	.406	0.002	0.006	.760

*Note: N* = 1656; β, probit coefficient; SE, standard error; *P*, probability; abuse, any physical or sexual abuse; N/A, not applicable. Bold signifies the positive results clear in the table.

## Discussion

### Main Findings

To the best of our knowledge, this is the first study to test the prospective association between adolescent cannabis use and hypomania in early adulthood, while controlling for a range of environmental risk factors, other substance use, and associated psychopathologies. Our analysis indicated 3 pathways whereby cannabis use is associated with subsequent hypomania.

First, adolescent cannabis use is independently associated with hypomania in early adulthood. This effect was independent of other key risk factors (or markers) for hypomania, including other drugs use, hazardous alcohol use, psychotic symptoms, and depression in late adolescence. This finding is consistent with population data in adults suggesting the association between cannabis use and manic symptomatology is independent of psychotic symptoms.^34^ The current study extends this previous work by demonstrating a prospective association in adolescents in which the cause (cannabis) is most likely to have preceded its effect (hypomania). The logistic regression analysis implied a dose–response relationship in that the odds ratios for *frequent use* was greater than for *any use*. Though we are tentative in our conclusions because of the observational nature of the data, our control for numerous confounders, the prospective association, and the dose–response findings are consistent with a causal role for cannabis use in the emergence of hypomania in young people.

Second, male sex is significantly indirectly associated with hypomania through an increased likelihood of cannabis use. Harmful use of cannabis is more frequent in men than women,^2^ and this study shows its use in men may be much more important in the pathway to hypomania.

Finally, childhood physical or sexual abuse is indirectly associated with hypomania through an increased likelihood of cannabis use. Both childhood abusive experiences and cannabis use in adolescence and adulthood have been shown to be connected to the expression of bipolar disorder psychopathology and to markers of its severity.^12,26,35^ Furthermore, previous studies have demonstrated that childhood trauma and cannabis use are also linked,^18^ with some studies indicating a prospective association independent of markers of deprivation or family dysfunction.^36^ The prospective analysis presented here is the first to show that cannabis use in adolescence may be part of the mechanism whereby childhood abuse can lead to bipolar disorder psychopathology. It appears that cannabis use seems to be one response of an individual to traumatic childhood experiences that can lead to harmful psychopathology in young adulthood. Our results align with those of Aas et al^18^ in highlighting the importance of both cannabis use and childhood trauma in bipolar disorder symptomatology.

The precise neurochemical mechanism by which cannabis might lead to hypomania is unclear, reflecting the incomplete knowledge of the biology of bipolar disorders in general.^37^ However, current evidence provides tentative insights. Neurobiological studies indicate the brain is particularly vulnerable to the effects of cannabis use during adolescence. It has the potential to adjust reward system sensitivity^38^ and can interfere with mechanisms related to establishing axonal connections during development. A recent systematic review of the literature highlights that converging evidence from imaging as well as pharmacological studies indicates a state of hyperdopaminergia in mania.^39^ Cannabis causes signaling changes in the meso-limbic system, resulting in dopaminergic hyperactivity, and some cannabinoid receptors are known to reduce the uptake of dopamine thereby potentiating its actions.^40^ Increased reward circuitry activity demonstrated by reduced differences in activation of dopaminergic brain areas (eg, nucleus accumbens) during anticipation and receipt of rewards has been demonstrated in manic patients; this differentiates them from patients with schizophrenia and healthy controls.^41^ Finally dopaminergic signaling is known to increase as part of normal brain maturation during the adolescent period.^42^ Cannabis use may compound this, leading to an increased propensity to experience hypomanic symptomatology.^15^

### Strengths and Limitations

This study had several strengths. The prospective assessments and sampling adolescents at age 17 years reduces the risk of reverse causality. The path analysis also controls for all associations between correlated items, enabling an examination of simultaneous pathways and determining the specificity of associations.

There are also study limitations. Our conclusions regarding the link between cannabis use and hypomania must remain cautious. The HCL-32 asks about a lifetime history of hypomania symptoms, and so in some cases the experience assessed at age 22–23 years may have preceded cannabis use. However, as the peak incidence of hypomania/mania appears to be bi-modal with the first peak between age 21 and 23 years and the second at around age 35 years,^16,43^ a majority of participants are likely to have first experienced hypomania after the age of cannabis use assessment. There may also be pre-existing psychopathology that could link to cannabis use and hypomanic symptoms that we did not account for.

The HCL-32 was used as a measure of hypomania but this will not always equate with a clinical diagnosis of hypomania. Nevertheless, we used a well-recognized cut-off score for hypomania, amplified by measures of duration and impact on functioning. This is likely to have improved the capacity of the HCL to identify clinical levels of hypomania.^23^ If they persist and cause dysfunction hypomanic symptoms are known to be continuous with and to have strong predictive values for diagnostic bipolar disorders.^44^ Finally, there was a high rate of attrition within the sample, but we carefully weighted our analysis according to predictors of drop-out.

Experiences of physical and sexual abuse were reported by the mother rather than child, potentially leading to an under-reporting of these factors. Under-reporting likely leads to “nondifferential misclassification,” ie, an under-reporting in both groups, which may exert a downward bias on estimated associations between abuse and outcomes.^45^ Data on childhood maltreatment between age 7 and 17 (when cannabis use data were collected) was not available and this may have impacted on our final model. Nevertheless, we still found a significant indirect association between abuse and hypomania via cannabis use suggesting this finding is relatively robust.

It is conceivable that hypomania symptoms may have been secondary to continuing use of substances such as cannabis and stimulants between assessment at age 17 and 22–23 years, though we do not know the extent to which use continued over this period. The HCL-32 requires people to consider a period in their life when they were feeling high while *not* using drugs or alcohol. This caveat in the questions in the HCL reduces the risk that hypomanic symptoms were closely linked to periods when a young person was using cannabis, stimulants, or alcohol.

## Conclusions

Despite these limitations, we show adolescent cannabis use is an independent risk factor for future hypomania, and the nature of the associations found is suggestive of a causal link, though the gold standard for inferring causality of course remains intervention. The study also identifies cannabis use as a candidate mechanism for explaining how childhood abuse may lead to hypomania in adulthood. This is important in the context of the push in many jurisdictions to legalize or decriminalize cannabis. Consistent with the stage specific model of mental disorders linking intervention to stage of illness,^46^ our findings suggest frequent cannabis use is likely to be a suitable target for interventions that may allay the risk of young people developing bipolar disorder.

## Funding

The UK Medical Research Council and Wellcome (102215/2/13/2) and the University of Bristol provide core support for ALSPAC.

## Supplementary Material

Supplementary-Table-1
