# Supplementary material for: Cannabis Use and Hypomania in Young People: A Prospective Analysis
Source: Schizophr Bull. 2017 Nov 28;44(6):1267–74. doi: 10.1093/schbul/sbx158 (PMC6192498; doi:10.1093/schbul/sbx158)
Supplement: Supplementary-Table-1 [file sbx158_suppl_supplementary-table-1.docx]

| Supplementary table 1. Drop-out analysis comparing those not available to those who completed the hypomania symptoms questionnaire | | | |
| --- | --- | --- | --- |
| Predictors | Completed questionnaire  N (%) | Did not complete questionnaire  N (%) | Non-response versus response  OR (95% CI) |
| Gender |  |  |  |
| Female | 2182 (30.2%) | 5037 (69.8%) | [reference] |
| Male | 1188 (15.6%) | 6447 (84.4%) | 0.43 (0.39, 0.46) |
| Birthweight |  |  |  |
| >2499g | 3020 (23.1%) | 1063 (76.9%) | [reference] |
| <2500g | 153 (18.8%) | 659 (81.2%) | 0.77 (0.65, 0.93) |
| Family adversity  (Mean score) | 3.42 (3.62) | 4.24 (4.22) | 0.95 (0.94, 0.96) |
| Childhood abuse |  |  |  |
| No | 2928 (26.9%) | 7976 (73.1%) | [reference] |
| Yes | 363 (26.7%) | 996 (73.3%) | 0.99 (0.87, 1.13) |
| Maternal hitting |  |  |  |
| No | 1615 (27.8%) | 4184 (72.2%) | [reference] |
| Yes | 1486 (27.5%) | 3908 (72.5%) | 0.99 (0.91, 1.07) |
| Maternal shouting |  |  |  |
| No | 1732 (28.6%) | 4317 (71.4%) | [reference] |
| Yes | 1369 (26.5%) | 3789 (73.5%) | 0.90 (0.83, 0.98) |
| OR=Odds Ratio; CI=Confidence Intervals; Significant results presented in bold type face. Family adversity assessed with the family adversity index which comprises 18 items (see Winsper et al. 2012 for more details). | | | |
